# Association Pattern of Interleukin-1 Receptor-Associated Kinase-4 Gene Polymorphisms with Allergic Rhinitis in a Han Chinese Population

**DOI:** 10.1371/journal.pone.0021769

**Published:** 2011-06-30

**Authors:** Yuan Zhang, Xiaoping Lin, Martin Desrosiers, Wei Zhang, Na Meng, Liping Zhao, Demin Han, Luo Zhang

**Affiliations:** 1 Key Laboratory of Otolaryngology, Head and Neck Surgery, Ministry of Education of China, Beijing Institute of Otorhinolaryngology, Beijing, People's Republic of China; 2 Department of Otolaryngology, Head and Neck Surgery, Beijing TongRen Hospital, Capital Medical University, Beijing, People's Republic of China; 3 Center of Allergy and Immunotherapy, The General Hospital of Shenyang Military Command, Shengyang, Liaoning, People's Republic of China; 4 Department of Otolaryngology, Hotel-Dieu Hospital, University of Montreal, Montreal, Quebec, Canada; 5 Department of Otolaryngology-Head and Neck Surgery, Montreal General Hospital, McGill University, Montreal, Quebec, Canada; Hospital of the University of Pennsylvania, United States of America

## Abstract

**Objective:**

Interleukin-1 receptor-associated kinase-4 (IRAK-4) encodes a kinase that is essential for NF-kB activation in Toll-like receptor and T-cell receptor signaling pathways, indicating a possible crosstalk between innate and acquired immunities. We attempted to determine whether the polymorphisms in the Interleukin-1 receptor-associated kinase-4 (IRAK-4) gene are associated with allergic rhinitis (AR) in the Han Chinese population.

**Methods:**

A population of 379 patients with AR and 333 healthy controls was studied. Blood was drawn for DNA extraction and total serum immunoglobulin E (IgE). A total of 11 single nucleotide polymorphisms (SNPs) in IRAK-4 were selected and individually genotyped.

**Results:**

Significant allelic differences between cases and controls were obtained for the SNP of rs3794262 in the IRAK-4 gene. In the stratified analysis for gender, two SNPs (rs4251431 and rs6582484) in males appeared as significant associations. Subgroup analysis for the presence of different allergen sensitivities displayed associations only in the house dust mite-allergic cohorts (rs3794262, rs4251481). None of the selected SNPs in IRAK-4 was associated with total IgE level. The haplotype analyisis indicated GCCTGCGA was significantly associated with AR. The SNP-SNP interaction information analysis indicated that the selected sets of polymorphisms had no synergistic effect.

**Conclusions:**

Our findings did not support the potential contribution of the IRAK-4 gene to serum IgE levels. However, the results demonstrated a gender- and allergen-dependant association pattern between polymorphisms in IRAK-4 and AR in Chinese population.

## Introduction

Allergic rhinitis (AR) is an inflammatory disease of the nasal mucosa induced by an immunoglobulin E (IgE)-mediated reaction in allergen-sensitized subjects. It is a disease of high prevalence, especially in industrialized countries, and has exhibited a fairly rapid upward trend [1]. Recent data on self-reported AR in the centre of cities across mainland China demonstrated a prevalence of 8.7%–24.1% [2]. This high prevalence translates into a high cost to society in terms of overall healthcare use and the quality of life of those with moderate-to-severe disease. The reasons for these trends remain unclear but probably reflect environmental influences on genetic predisposition. AR has multifactorial inheritance, and it is likely that different combinations of genes or single nucleotide polymorphisms (SNPs) increase the risk of phenotypic expression, resulting in allergic inflammation. Another explanation is proposed in the hygiene hypothesis, which suggests that as a result of modern public health practices, individuals encounter a reduced microbial burden, rendering them vulnerable to the development of allergic disease [3].

Interleukin-1 receptor-associated kinase-4 (IRAK-4) was selected as a candidate gene for AR based on its central function in innate and acquired immunity [4]. The human body is protected against external pathogens by two immune systems: innate and acquired immunities. Whereas innate immunity exhibits immediate responses to external pathogens by recognizing pathogen-associated molecular patterns (PAMPs), acquired immunity uses T cells to recognize and defend against pathogens by developing effector cells, antibodies and memory cells. Although each system seems to possess distinct activation mechanisms, there could be crosstalk between these two signaling pathways. IRAK-4 is essential for NF-kB activation in Toll-like receptor (TLR) and T-cell receptor (TCR) signaling pathways, suggesting that IRAK-4 may be involved in direct signal crosstalk between the two systems [5]. TLRs have been identified as sensors for invading microbes and as key players in the initiation of Th1 immune responses. Therefore it is conceivable that weak TLR stimulation might influence the pathogenesis of allergic disease since weak Th1 imprinting may result in unrestrained Th2 responses. On the other hand, as T cells express several TLRs [6], [7], it is possible that TLR ligands could influence T-cell function directly. Moreover, TLRs on regulatory T (Treg) cells have also been suggested to modulate acquired immune responses by regulating the suppressive functions of Treg cells [8], [9] which were crucial players in diseases characterized by dysregulated peripheral tolerance including allergic diseases [10].

Given the evidence above, we hypothesized that IRAK-4 may be a strong candidate gene for AR and that SNPs in the IRAK-4 gene region may influence the risk of developing AR. Therefore, the aim of this study was to examine whether and how polymorphisms in the IRAK-4 gene are associated with susceptibility to develop AR. A population-based case-control association analysis was used to assess the risk of AR conferred by SNPs in the IRAK-4 gene region in our Han Chinese cohort.

## Materials and Methods

### Study subjects

Three hundred and seventy-nine affected individuals (227 males and 152 females) with AR were prospectively recruited from the rhinology clinic and ward of Beijing Tongren Hospital and the Allergy and Immunotherapy Center of The General Hospital of Shenyang Military Command from February 2008 to July 2009. A total of 333 controls who were healthy volunteers were recruited from an ethnically similar local population to determine similar background population allele frequencies. All subjects were of Han Chinese ethnic origin and all from the northern region of China. The study was approved by the Ethics Committees of Beijing TongRen Hospital and The General Hospital of Shenyang Military Command, and written informed consent was obtained from all participants.

Patients were diagnosed as AR and included if they tested positive for all the following criteria in terms of the ARIA (Allergic Rhinitis and its Impact on Asthma, 2008) [11] guideline. 1) persistent or discontinuous symptoms of anterior rhinorrhea, continuous sneezing, nasal obstruction and itching, 2) nasal endoscopy showed a pale and edematous nasal mucosa, nasal discharge and swollen inferior turbinates and 3) positive serum antigen-specific IgE as measured by the ImmunoCAP 100 system (Pharmacia, Uppsala, Sweden) or positive antigen skin prick test (SPT) (Allergopharma, Reinbeck, Germany). Last but not least, a diagnosis of AR was obtained from the presence of symptoms when exposed to the allergen in question in conjunction with a positive skin test response to the same allergen. The healthy controls presented no clinical features, local nasal cavity signs, family history of allergic disease and showed negative of serum antigen-specific IgE phadiatop determination.

Diagnosed AR individuals meeting the following criteria were excluded from the study. (1) AR combined with hypertension, diabetes or other chronic diseases; (2) AR combined with tumor in the nasal cavity; (3) AR combined with eczema or asthma.

The antigen included house dust mite (HDM) (Der f and Der p); seasonal grass pollens (Gaint Ragweed; Mugwort; Lamb's quarers; Humulus; Chenopodium album and so on); animal Hair (especially dog and cat); molds (in door and out door mustiness or floricultural environment) and cockroach. Subjects were considered to be sensitive to allergens if the measurement of serum IgE was equal to or above 0.35 kU/l. A positive SPT result was defined as a wheal greater than or equal to one half of the diameter of the histamine control and at least 3 mm larger than the diameter of the negative control [12]. We defined mixed allergen allergy to be when an individual showed positive results to two or more allergens in the skin test or serum examination. Serological and skin testing were performed by specialist technicians and nurses respectively, while the AR diagnoses were made by clinical rhinologists.

### Selection of polymorphisms in the human IRAK-4 gene

We choose the majority of the tag SNPs (tSNPs) from the Hapmap database and the selection strategy was as follows: Firstly, the International Haplotype Mapping (HapMap) (www.hapmap.org) SNP databases were used to select tSNPs in the IRAK-4 gene region and the screened region was extended 10 kilobases upstream of the annotated transcription start site and downstream at the end of the last IRAK-4 exon. The tSNPs were selected to extract most of the genetic information in the region using the CHB genotyping data from the HapMap database (HapMap data rel 27 Phase II+III, Feb2009) [13]. From this dataset, genotyping data for 34 tSNPs were obtained and loaded in the Haploview software version 4.1 [14] (Supporting information, Figure S1). Secondly, tSNPs were then selected using a pairwise tagging algorithm setting the Hardy-Weinberg p value, minor allele frequency (MAF) and r2 thresholds at 0.01, 0.05 and 0.8, respectively. The linkage disequilibrium (LD) pattern of the IRAK4 gene in the CHB population exhibited strong LD in several groups of tSNPs (r^2^ greater than or equal to 0.8) as displayed in Supporting information Figure S1, indicating that most common SNPs can be captured by a subset of tagging SNPs. Consequently, we choose 10 SNPs, including rs4251513, rs1461567, rs3794262, rs4251481, rs4251540, rs4251569, rs6582484, rs4251431, rs1870765 and rs12302873 to represent the entire 34 loci for eventual genotyping. Moreover, one additional SNP (rs4251559) from the previous literature was also included [15]. Therefore, 11 SNPs constituted the selection set to be genotyped in our patients and controls.

### Single nucleotide polymorphism genotyping

DNA was isolated from peripheral blood leukocytes and collected in EDTA-treated tubes, using the DNA Isolation Kit for Mammalian Blood(Roche, Indianapolis, USA). Isolated DNA from blood was stored at 4°C for less than 2 days prior to use. The majority of the selected SNP genotyping was performed with the Sequenom MassARRAY iPLEX Gold platform (Sequenom, San Diego, California) according to the manufacturer's instructions. The polymerase chain reaction (PCR) and extension primers were designed using MassARRAY Assay Design 3.1 software (Supporting information, Table S1). One SNP (rs12302873), which was evaluated by preliminary testing as unsuitable for genotyping through the MassArray approach was identified by direct sequencing of the PCR products of genomic DNA (Supporting information, Table S1). Genotyping was performed without knowledge of the case or control status. A 10% random sample was tested in duplicate by different persons, and the reproducibility was 100%.

### Statistical analyses

Quality tests were processed using Haploview version 4.1 software for the first step to filter the data and decided which were suitable for the further statistical tests. Hardy-Weinberg equilibrium (HWE) of each SNP was assessed in controls only and a threshold *P*<0.001 was regarded to indicate deviation from HWE. In addition, we assessed the MAF, non-missing genotype percentage and other criteria in the AR cases as well as controls to filter the data. Among them, MAF and non-missing genotype percentage thresholds was set at <0.001 and <75% respectively. SPSS software version 13.0 was used to determine association. Chi-square tests were performed to determine whether an association existed for alleles and genotypes between cases and controls. Associations with susceptibility to AR were tested by calculating odds ratios (OR) with asymptotic 95% confidence intervals (95% CI), and *P* values less than 0.05 were considered statistically significant. Bonferroni correction over the tested SNPs of IRAK-4 gene gave an adjusted significance threshold in multiple testing.

Gender was taken into account to estimate whether there were differences between the two alleles for the contributions of risk. The Breslow-Day Test was performed to evaluate the homogeneity between genders. *P* values less than 0.05 was regarded as heterogeneity and male and female cohort should be assessed separately. Subanalysis restricted to the presence of different allergens - house dust mite, pollens and mixed allergens - was also performed to examine whether the effect of associations within the population differed among the subgroups. Associations between genotype and IgE levels for all AR and control subjects were assessed using an ANOVA test. Homogeneity of variance test was made using Levene test firstly. A threshold of *P* for Levene Statistic<0.05 was considered to indicate a non-normal distribution of IgE level. Kruskal-Wallis ANOVA was used for association of IRAK-4 SNPs with non-normal distribution IgE levels.

Haplotype association analysis was performed using SHEsis, a web-based software platform that allowed the estimation of haplotype frequencies (http://analysis.bio-x.cn/SHEsisMain.htm). We compared haplotype frequencies between AR patients and controls by Chi^2^-test from a series of 2×2 contingency tables by combining other haplotypes.

The evaluation of SNP-SNP interactions was performed using the four-step process outlined by Moore et al. [16], [17] using the multifactor dimensionality reduction (MDR) constructive induction algorithm [18], [19]. A single best model was selected that maximized the testing accuracy. The genetic data were collapsed into two categories, high and low risk by exhaustively searching all single locus and multilocus combinations of the data and then categorizing each multilocus genotype cell into either high risk or low risk on the basis of the ratio of AR and control subjects in each cell. Interactions dendrograms were used to visualize the nature of the dependencies. All analyses were implemented in the open-source MDR software package (v.3.3.0) available from http://faculty.washington.edu/browning/beagle/beagle.html.

The statistical power for the present study was calculated using G*Power 2 software (http://www.psycho.uni-duesseldorf.de/aap/projects/gpower/). When the parameter was set on 0.2 which represented the effect of the allele of the gene was minor, the power of the present overall and subgroup collected sample size as regarding the association study was evaluated [20].

## Results

### Population characteristics

The characteristics of the study population are shown in Table 1. Age and gender were all well-balanced between cases and controls. The cohort of 379 AR patients had a mean age of 27 years and consisted of more men (59.9%) than women (40.1%), while the 333 control individuals had a mean age of 36 years and a similar component of more men (53.8%) than women (46.2%). The mean total serum IgE measurements for the case and control groups were 283.1±512.7 and 68.3±119.2 IU/ml respectively. Patients were diagnosed as having AR by combining the skin test and serum specific IgE data with the allergen-specific case history and nasal physical examination. 73.9% of individuals demonstrated perennial nasal symptoms while the remainder reported seasonally-related reactions. When subjects were classified according to the serum allergen-specific IgE category, 216 (57%), 64 (16.9%) and 99 (26.1%) were allergic to HDM, pollens and mixed allergens respectively.

**Table 1:** Demographic characteristics of the study population.

Characteristic	AR cases (n = 379)	Controls (n = 333)
Age Mean (Range) (years)	26.9±14.8 (2–71)	36.3±15.3 (3–78)
Sex, M/F, No. (%)	227 (59.9)/152 (40.1)	179 (53.8)/154 (46.2)
Total IgE, kU/l	283.1±512.7	68.3±119.2
Perennial/seasonal, No.(%)	280 (73.9)/99(26.1)	-
Allergen category, No. (%)		
** **House dust mite	216 (57.0)	-
** **Pollens	64 (16.9)	-
** **Mix	99 (26.1)	-

-: no results.

### Descriptive analysis

**Figure 1:**
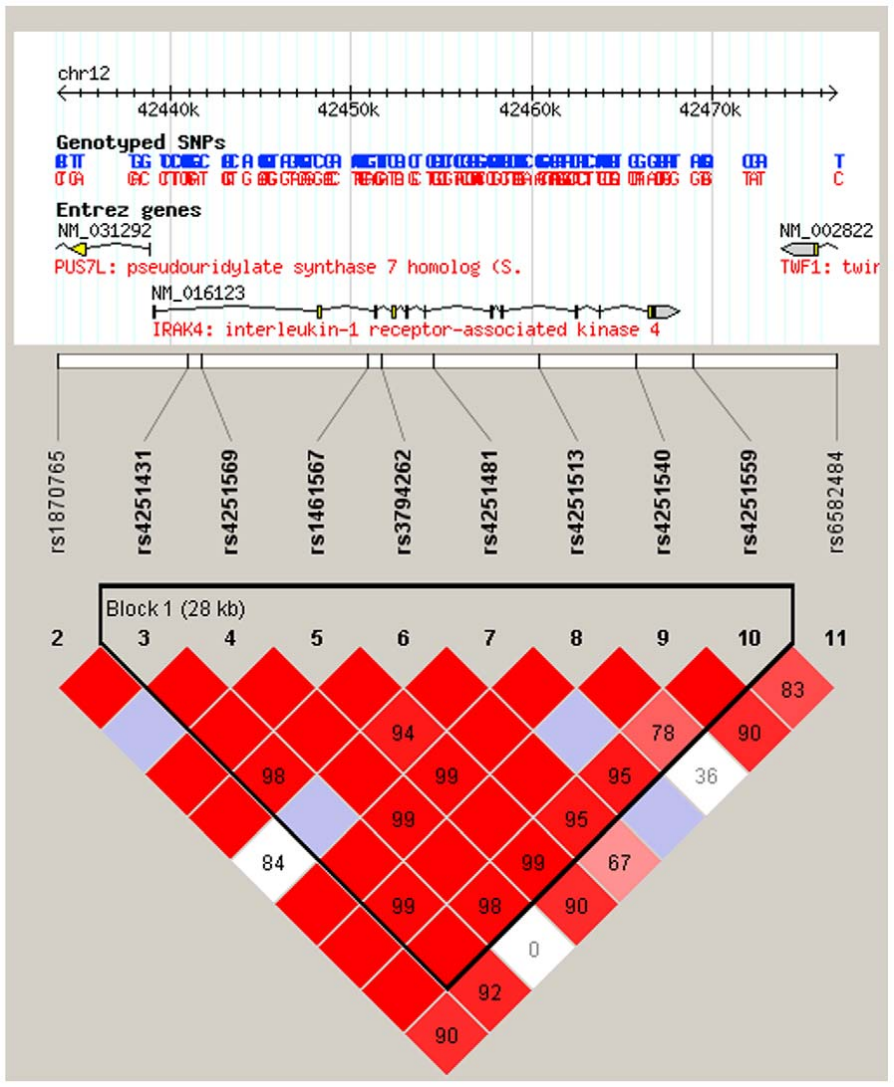
Linkage Dysequilibrium (LD) plot for IRAK-4. The LD plots were generated by Haploview 4.1. The upper part of the graph illustrates the HapMap info track including the location of the gene on the chromosome. The white horizontal bar below the info track illustrates the location of SNPs on a physical scale. The value within each diamond represents the pairwise correlation between tagging SNPs defined by the upper left and the upper right sides of the diamond. Shading represents the magnitude and significance of pairwise LD, with a red-to-white gradient reflecting higher to lower LD values.

The quality testing results of the SNPs in the IRAK4 gene selected for genotyping were shown in supporting information Table S2. All SNP loci examined were in Hardy-Weinberg equilibrium (HWE). One SNP (rs12302873) demonstrated bad quality in terms of the low non-missing genotype percentage (71.1%) among the entire samples. The data for this locus was excluded for further analysis. The Linkage Dysequilibrium (LD) plot of the remaining genotyped SNPs surrounding the IRAK4 gene in our population collected in present study, as generated by Haploview version 4.1 is displayed in Figure 1, tagging one block. The statistical power of the entire sample size (379 cases VS 333 controls) in this study was 88.45% as regards the association analysis. The power of the subgroup analysis as for the gender was 60.79% (Male) as well as 45.95% (Female), and 76.88% (House dust mite), 59.42% (Pollens) and 64.19% (Mix) respectively regarding the different allergen categories.

### Association analysis

**Figure 2:**
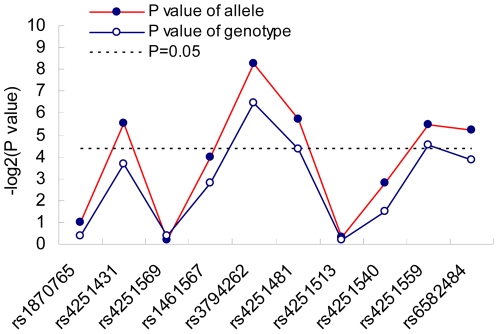
Significance of each tagging SNP. The *x* axis shows the genomic position, and the *y* axis shows the negative base-2 logarithm of the *P* values for each allele and genotype of each SNP. Lines are drawn between points only for the sake of clarity, as the disease association between markers is not linear.

The analysis of allele frequencies for the remaining 10 SNPs tested, shown in Table 2, revealed significant associations (*P*<0.05) with an AR outcome in the IRAK-4 gene at 5 loci (rs4251431, rs3794262, rs4251481, rs4251559 and rs6582484). Only one SNP (rs3794262, *P* = 0.0033, OR = 1.5764) remained significant following application of the Bonferroni correction of multiple testing for 10 tests (*P*<0.005), indicating allele A was a risk factor for AR susceptibility. Meanwhile, results for the analysis of association between the related genotypes of each SNP and AR were also obtained, again showing associations in 3 loci (rs3794262, rs4251481 and rs4251559) (Table 2). However, no SNP attained the threshold required after application of Bonferroni correction for 10 tests (p<0.005). The P values for the allele and genotype of each SNP illustrating according to the sequence of the genomic position were plotted in Figure 2.

**Table 2:** Allele and genotype frequencies and AR susceptibility.

SNP	Minor allele	Case, Control Frequencies	Allele *P*	OR(95%CI)	Genotype *P*	OR_Het_ (95%CI)	OR_Hom_ (95%CI)
rs1870765	C	0.0179, 0.0231	0.4924	1.2997 (0.6137–2.7525)	0.7861	0.7651(0.3584–1.6333)	-
s4251431	T	0.0666, 0.1019	0.0216	1.5917 (1.0678–2.3726)	0.0805	0.6300(0.4034–0.9840)	0.5445 (0.1290–2.2976)
rs4251569	T	0.1556, 0.1528	0.8831	0.9782 (0.7295–1.3118)	0.7573	0.9602(0.6870–1.3419)	1.5730 (0.4562–5.4235)
rs1461567	T	0.5124, 0.4623	0.0649	1.2226 (0.9876–1.5134)	0.144	1.0997(0.8134–1.4869)	1.2448 (0.8671–1.7868)
rs3794262	T	0.1171, 0.1729	0.0033	1.5764 (1.1620–2.1386)	0.0115	0.6195(0.4369–0.8785)	0.5464 (0.1769–1.6876)
rs4251481	G	0.0442, 0.0746	0.019	1.7427 (1.0903–2.7854)	0.049	0.5841(0.3585–0.9518)	-
rs4251513	G	0.3705, 0.3638	0.7959	0.9714(0.7796–1.2104)	0.8605	1.0870(0.8050–1.4679)	0.9685 (0.6214–1.5095)
rs4251540	T	0.0732, 0.0950	0.1458	1.3292 (0.9050–1.9524)	0.359	0.7545(0.4932–1.1542)	0.6623 (0.1471–2.9816)
rs4251559	G	0.5627, 0.5017	0.023	0.7719(0.6175–0.9650)	0.044	1.1810(0.8610–1.6200)	1.2309(0.8731–1.7353)
rs6582484	C	0.0809, 0.4983	0.0264	1.5076(1.0474–2.1700)	0.0688	0.6186(0.4058–0.9431)	0.7891(0.2623–2.3734

OR_Het_ :odds ratios for heterozygotes OR_Hom_ : odds ratios for homozygotes.

-: show no case of homzygotes.

Likewise, stratifying the AR cohort and controls by gender succeeded in detecting evidence of association between the SNP markers and AR, which appeared to show differences between males and females (Table 3). The allele model association analysis demonstrated that 4 SNPs (rs4251431, rs3794262, rs4251540 and rs6582484) in males and 3 SNPs (rs1461567, rs4251481 and rs4251559) in females were associated with AR respectively, indicating a gender-dependant association pattern between polymorphisms in IRAK-4 and AR. While only two SNP (rs4251431, *P* = 0.0030, OR = 1.5196; rs6582484, *P* = 0.0024, OR = 1.4692 ) in males attained the threshold required after application of Bonferroni correction (*P*<0.005). The Breslow-Day Test for homogeneity among different gender cohorts showed only one SNP (rs6582484, *P* = 0.044) presented the heterogeneity among males and females, suggesting data analysis for this locus should be performed separately. Consistently, rs6582484 showed significant association with AR susceptibility in males (*P* = 0.0024, OR = 1.4692) while no association in female cochort (*P* = 0.9253).

**Table 3:** Male and female cohort homogeneity test and allele model association analysis.

SNP	Breslow-Day Test	Males		Females	
	*P*	*P*	OR (95%CI)	*P*	OR (95%CI)
rs1870765	0.908	0.6547	1.1123 (0.6785–1.8230)	0.5919	1.1876 (0.6050–2.3313)
rs4251431	0.068	0.0030	1.5196 (1.0982–2.1028)	0.9312	1.0140 (0.7379–1.3935)
rs4251569	0.866	0.7734	0.9742 (0.8169–1.1617)	0.9682	0.9956 (0.8018–1.2362)
rs1461567	0.259	0.5180	0.9595 (0.8465–1.0876)	0.0390	1.1868 (1.0082–1.3969)
rs3794262	0.602	0.0073	1.2981 (1.0513–1.6028)	0.1354	1.2195 (0.9233–1.6108)
rs4251481	0.187	0.3107	1.1504 (0.8587–1.5411)	0.0124	1.7957 (1.0295–3.1321)
rs4251513	0.082	0.1667	0.9130 (0.8040–1.0367)	0.2716	1.1003 (0.9259–1.3076)
rs4251540	0.057	0.0170	1.3495 (1.0206–1.7845)	0.5862	0.9194 (0.6870–1.2305)
rs4251559	0.462	0.2392	1.0828 (0.9477–1.2372)	0.0443	1.1978 (1.0032–1.4301)
rs6582484	0.044	0.0024	1.4692 (1.1011–1.9604)	0.9253	0.9832 (0.7393–1.3157)

As regarding the subgroup analysis for the presence of sensitivity to different allergens displayed significant association only in HDM allergic cohorts (rs1461567, rs3794262, rs4251481 and rs4251559) (Table 4), while it failed to detect evidence of association in the other two subgroups (data not shown). Among the four SNPs, rs3794262 (*P* = 0.0034, OR = 1.7388) and rs4251481 (*P* = 0.0023, OR = 2.6593) remained significant following application of the Bonferroni multiple testing.

**Table 4:** Allele and genotype frequencies and house dust mites AR susceptibility.

SNP	Minor allele	Case, Control Frequencies	Allele *P*	OR(95%CI)	Genotype *P*	OR_Het_(95%CI)	OR_Hom_(95%CI)
rs1870765	C	0.0195, 0.0231	0.6928	1.1908(0.5003–2.8341)	0.9233	0.8365(0.3482–2.0098)	-
rs4251431	T	0.0741, 0.1019	0.1386	1.4189(0.8914–2.2586)	0.3181	0.7705(0.4632–1.2814)	0.3234(0.0375–2.7896)
rs4251569	T	0.1585, 0.1528	0.8009	0.9571(0.6808–1.3456)	0.5648	0.9388(0.6340–1.3903)	2.0000 (0.5307–7.5366)
rs1461567	T	0.5417, 0.4623	0.0123	1.3748(1.0713–1.7643)	0.0289	1.1493 (0.8079–1.6351)	1.4094 (0.9342–2.1262)
rs3794262	T	0.1073, 0.1729	0.0034	1.7388(1.1966–2.5267)	0.0124	0.5767 (0.3788–0.8781)	0.3855(0.0810–1.8335)
rs4251481	G	0.0294, 0.0746	0.0023	2.6593(1.3865–5.1007)	0.0087	0.3765 (0.1930–0.7346)	-
rs4251513	G	0.3537, 0.3638	0.7385	1.0450(0.8072–1.3529)	0.7541	1.0907 (0.7681–1.5486)	0.8229 (0.4797–1.4116)
rs4251540	T	0.0780, 0.0950	0.3445	1.2402(0.7932–1.9392)	0.5655	0.8668 (0.5328–1.4102)	0.3885 (0.0431–3.5004)
rs4251559	G	0.5870, 0.4983	0.0075	1.4307(1.1001–1.8605)	0.0203	1.1867 (0.8202–1.7170)	1.3687 (0.9217–2.0325)
rs6582484	C	0.0821, 0.1172	0.0731	1.4851(0.9617–2.2933)	0.1996	0.6532 (0.3969–1.0751)	0.6987 (0.1785–2.7341)

In addition, none of the 10 SNPs in the IRAK-4 gene was associated with serum total IgE level (Table 5).

**Table 5:** Association between IRAK-4 SNPs and IgE levels.

SNP	*P* for Levene Statistic	F	*P*
rs1870765	0.512	0.053	0.818
rs4251431	0.236	0.862	0.423
rs4251569	0.216	0.745	0.475
rs1461567	0.025	0.543*	0.762*
rs3794262	0.164	0.909	0.404
rs4251481	0.396	0.278	0.598
rs4251513	0.002	1.784*	0.41*
rs4251540	0.268	0.86	0.424
rs4251559	0.014	0.737*	0.692*
rs6582484	0.257	0.596	0.551

*: results using Kruskal-Wallis ANOVA.

### LD and haplotype analysis

To assess the extent of linkage disequilibrium (LD) across the IRAK-4 gene, we calculated LD values. Some of the pair-wise LD values in the IRAK-4 gene were close to 1 among all SNPs, indicating very strong LD. As the SNPs were in only one LD blocks (Figure 1), the haplotypes of the markers of the IRAK-4 gene were analyzed accordingly. Among all the haplotypes which were predicted, only 6 had a frequency above 1%. Loosely, the omnibus haplotype test showed one haplotype (GCCTGCGA) was associated with AR (*P* = 0.0483) and the frequency of GCCTGCGA was significantly lower (4.4% vs. 6.9%) in AR than that in controls (Table 6). However this haplotype could not reach the significant level *P*<0.005 (0.05/10) when using the stringent Bonferroni correction.

**Table 6:** Haplotype frequencies and AR susceptibility.

Haplotype	Case Frequencies	Control Frequencies	df	Chi Square	*P*
GCTAACGG	0.510	0.462	1	3.036	0.0814
GCCAAGGA	0.171	0.178	1	0.116	0.7329
GTCAAGGA	0.152	0.153	1	0.008	0.9297
TCCTACTA	0.073	0.097	1	2.473	0.1159
GCCTGCGA	0.044	0.069	1	3.899	0.0483
GCCAAGGG	0.043	0.034	1	0.688	0.4069

*df*: degree of freedom.

### SNP-SNP synergistic analysis

Supporting information Figure S1 summarized the interaction information analysis of the selected polymorphisms. Shown is an interaction dendrogram highlighting the amount of information gained about case-control status by putting five polymorphisms together using the MDR function. The single best MDR model was selected in terms of the maximized the testing accuracy (0.5559) (Shown in supporting information Table S3). The interaction information analysis indicated that the selected sets of polymorphisms had no synergistic effect.

## Discussion

In this study, we aimed to evaluate the contribution of single nucleotide polymorphisms (SNPs) in the IRAK-4 gene region to allergic rhinitis (AR) susceptibility in a Chinese population-based case-control association analysis. Significant allelic differences between cases and controls were obtained for rs3794262 in the IRAK-4 gene. As regards the stratified analysis for gender, two SNPs (rs4251431 and rs6582484) in males appeared to be significant associations. Subgroup analysis for the presence of different allergen sensitivities displayed associations only in HDM allergic cohorts (rs3794262, rs4251481). Additionally, none of the selected SNPs in IRAK-4 was associated with total IgE level. The haplotype analyisis indicated GCCTGCGA was significantly associated with AR. The SNP-SNP interaction information analysis indicated that the selected sets of polymorphisms had no synergistic effect. Our study provides the first evidence for an association pattern of IRAK-4 polymorphisms with AR in a Chinese population.

Allergic sensitization is defined by production of IgE against environmental antigens such as house dust mite, grass pollen, and animal proteins and can lead to diseases that include asthma, rhinitis and atopic dermatitis [21]. Allergic disease prevalence rates have increased dramatically over the last 50 years in developed countries and one explanation might be that modern practices in public health lead to a decreased exposure to pathogens resulting in a misguided immune response [22]. The immunological explanation has been put into the context of the functional T cell subsets known as T helper 1 (Th1) and T helper 2 (Th2) that display polarized cytokine profiles. It has been argued that bacterial and viral infections during early life direct the maturing immune system toward Th1, which counterbalances pro-allergic responses of Th2 cells. Thus, a reduction in the overall microbial burden and low stimulation of Toll-like receptors (TLRs) will result in weak Th1 imprinting and unrestrained Th2 responses that presumably allow an increase in allergic responses. This concept is the basis for the hygiene hypothesis [23], [24].

It has become evident that immune responses to pathogens are initiated by TLRs that recognize a variety of structures derived from viruses, bacteria, fungi or protozoa [25], [26]. The molecules contributing to innate and acquired responses might not be strictly discrete but, instead, could overlap partially. It has been reported that the signaling pathways in adaptive immunity might share mediators with those in innate immunity [27]. Meanwhile, several reports also have described the crucial roles of TLRs expressed on T cells in adaptive immunity [28]–[30]. Both TLR- and T-cell receptor (TCR)-induced NF-kB activation in T cells are IRAK-4-dependent, there could be crosstalk between these two signaling pathways. This possible synergy in NF-kB activation would be crucial for optimizing T-cell functions [4], [5].

Several distinct mutations in IRAK-4 have been described in humans, with loss of function associated with dramatic and recurrent bacterial infections, as well as poor inflammatory response [31]–[33]. However, none of the reported mutations could be replicated in a different population, suggesting that these variants are uncommon. A recent study from Desrosier's group demonstrated a clear association between polymorphisms in the IRAK-4 gene and serum IgE levels in patients with chronic rhinosinusitis (CRS) and asthma, indicating IEAK-4 may be important in the regulation of IgE levels in patients with inflammatory diseases of the airways [15]. Here we did not support the potential contribution of the IRAK-4 gene to the serum IgE levels. The reason for the failure to replicate could be interpret as follows. Firstly, although both AR and CRS are upper airway inflammatory diseases and even there are some cross points in the respective pathogenesis, AR is clearly related to allergy while CRS is more related to the colonization by specific bacterial pathogens, indicating different disease mechanism that may potentially determined by different genetic background. Moreover, here we completely excluded the patients with atopic dermatitis and asthma, presenting an obvious contrast against Desrosier's study. In addition, this could be due to the population differences. It is known that a single SNP that is not a missense mutation might have only a small effect and might not efficiently discriminate between cases and control subjects in a genetic association allergy study. However, patterns of SNPs could contribute to the risk of a complex disease. SNP-SNP interactions could provide insight into the relationship between genes and their combined effect. It has been elucidated that SNP-SNP interactions between GATA3 and IL13 polymorphisms can influence the risk of childhood rhinitis [34]. Likewise, gene–gene interactions, that is, the functional interplay between genetic variants within a pathway, are also likely to contribute to the complexity of genetic diseases. Each variant typically has modest effects in isolation, but synergizes effectively with other variants to magnify the impact on disease risk [35]. Analyses of associations between allergy and variants in the Th2-cell differentiation and signaling pathway provide good examples of gene–gene interactions [36]. Although none of the selected SNPs for screening in the present study were in coding regions of IRAK-4 gene and we didn't find the SNP-SNP interactions of the IRAK-4 gene, we detected one haplotype (GCCTGCGA) was associated with AR (*P* = 0.0483) and the frequency was significantly lower (4.4% vs. 6.9%) in AR than that in controls. Further studies related to SNP-SNP or gene-gene interactions in the TLR and TCR pathway should be carried out to explore the mechanism of AR development.

Interestingly, here we reported two SNPs (rs3794262, rs4251481) showed a strong association with AR in patients allergic to HDM but not in patients allergic to pollens or mixed allergens. Actually, it has been demonstrated that HDM allergens could induce innate immune responses via TLR4 triggering of airway structural cells and TLR4 expression is necessary and sufficient for activation of immune responses by mucosal DCs and development of Th2 immunity and allergic inflammation [37]. HDM extract contains endotoxin and its levels in house dust have been correlated with a modifying effect on allergic sensitization in children [38]. Eisenbarth et al. proposed that low dose endotoxin promotes Th2 immunity, whereas hight dose promotes Th1 responses [39]. Recently, the relevant Der p 2 allergen was found to enhance the response of murine bronchial epithelial cells to endotoxin by acting as an MD2 like chaperone that promotes TLR4 signalling [40]. It will be interesting to study the possible link between the IRAK-4 gene, TLR4 signalling and the proallergic innate response to HDM AR.

Furthermore, it is important to note that the significance of the association detected in this study was not very strong and the statistical power of sample size in this study was only 88.45% as regards the association analysis. Future efforts to identify IRAK-4 SNPs carrying a smaller IRAK-4 risk will require a larger sample size than has been used here. Moreover, as with many other complex disorders, AR is thought to be the result of a complicated network of numerous susceptibility loci, many of which exert additive or synergistic effects, but have only a small role when considered in isolation [41], [42]. Among other reasons, failure to identify and replicate individual genetic risk factors for complex diseases has been attributed to epistasis obscuring the effect of single loci [43], [44]. Further studies including gene-gene and gene-environment interactions in AR cohorts are needed to clarify the impact of IRAK-4 on allergic disease.

Last but not lease, with the development of Genome-wide association studies (GWAS) till now several GWAS for asthma, total IgE and other allergy related phenotypes have been performed and some chromosome regions as well as specific genes have been implicated to be susceptible for the related phenotype in the certain population till now [45]–[48]. However there is no GWAS particularly for AR and moreover, no study reports a genetic association between IRAK-4 and allergy/atopy from GWAS approach. In addition, Sven Michel et al [49] recently pointed out that GWAS coverage was insufficient for many asthma candidate genes, imputation based on these data is reliable but incomplete. In this sense, we need conduct a more efficient combination for GWAS and current hypothesis driven approaches in the future.

In summary, the present findings revealed that IRAK-4 polymorphisms were associated with AR in a gender- and allergen-dependant pattern. Whereas, it is difficult for the candidate gene approach to provide insight into disease mechanism without replication in a second population and function study. Future studies will be mainly aimed at further elucidating the mechanisms underlying sensitization to different allergens and larger cohorts of patients with these conditions, in a variety of ethnic populations, are required to increase our knowledge-base for the IRAK-4 gene and may shed light on studies of the etiology of AR. Although the link between the innate and adaptive immune systems is well established, the importance of IRAK-4 in this respect remains to be fully understood. Studies that include additional genes and environmental factors in a systematic assessment will likely improve the understanding of the interactions of genes in the TLR pathway in rhinitis and associated phenotypes.

## Supporting Information

Figure S1 **Interaction dendrogram for the five polymorphisms modeled by the MDR method.** A red or orange line (shown no red or orange line in present study) connecting two polymorphisms suggests a positive information gain which can be interpreted as a synergistic or non-additive relationship while a blue or green line suggests a loss of information which can be interpreted as redundancy or correlation. A yellow line indicates independence or additivity.
(TIF)

Table S1 **Details of the primers used in the screening of SNPs by MassArray and PCR direct sequencing.**
(DOC)

Table S2 **SNPs in the IRAK-4 gene selected for genotyping and results of quality testing.**
(DOCX)

Table S3 **MDR analysis summary.**
(DOCX)
