# Supplementary figures and images for: Association Pattern of Interleukin-1 Receptor-Associated Kinase-4 Gene Polymorphisms with Allergic Rhinitis in a Han Chinese Population
Source: PLoS One. 2011 Jun 30;6(6):e21769. doi: 10.1371/journal.pone.0021769 (PMC3128076; doi:10.1371/journal.pone.0021769)

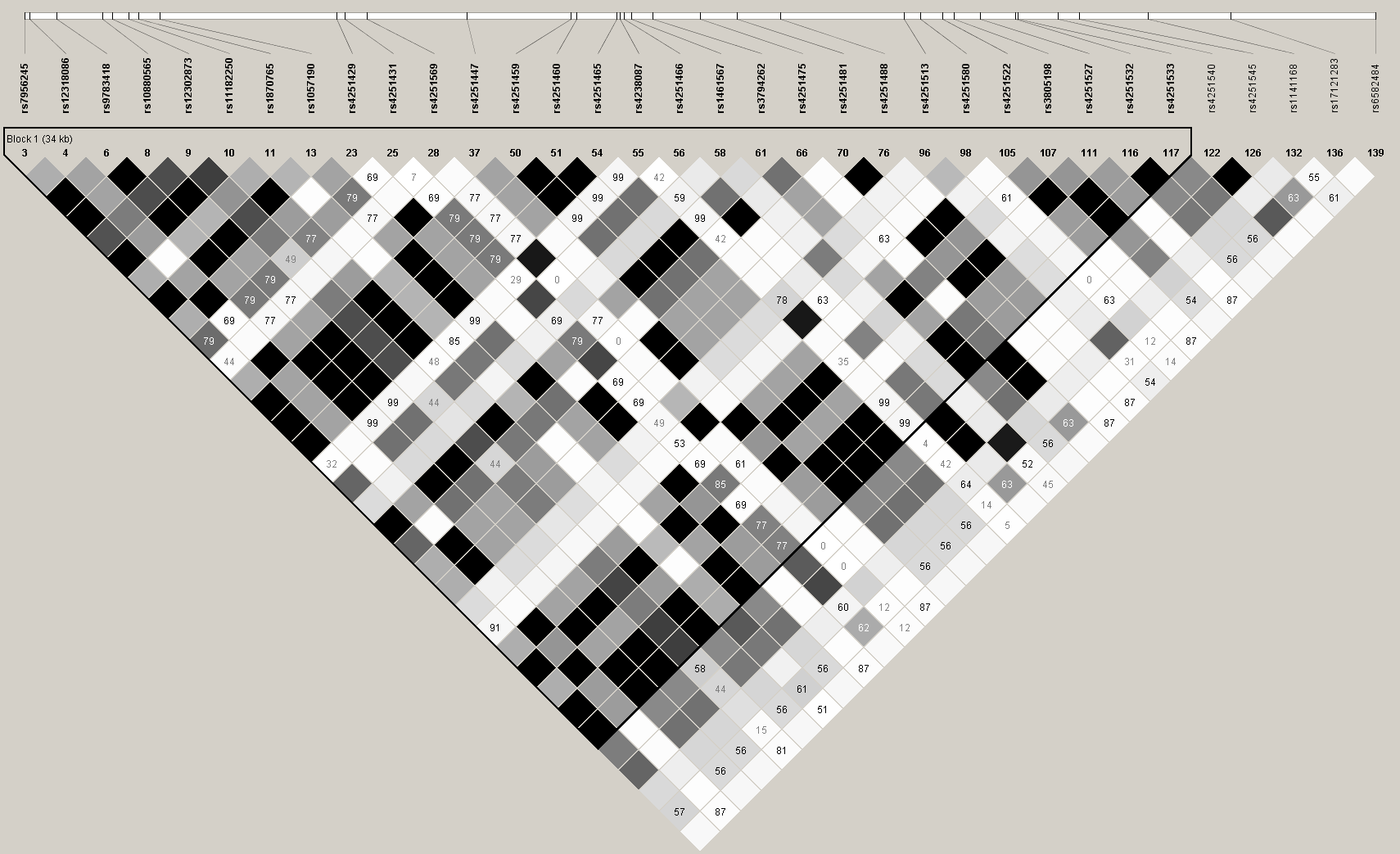

Supplement: Figure S1 — Interaction dendrogram for the five polymorphisms modeled by the MDR method. A red or orange line (shown no red or orange line in present study) connecting two polymorphisms suggests a positive information gain which can be interpreted as a synergistic or non-additive relationship while a blue or green line suggests a loss of information which can be interpreted as redundancy or correlation. A yellow line indicates independence or additivity. (TIF) [file pone.0021769.s001.tif]
