# Supplementary material for: Association Pattern of Interleukin-1 Receptor-Associated Kinase-4 Gene Polymorphisms with Allergic Rhinitis in a Han Chinese Population
Source: PLoS One. 2011 Jun 30;6(6):e21769. doi: 10.1371/journal.pone.0021769 (PMC3128076; doi:10.1371/journal.pone.0021769)
Supplement: Table S1 — Details of the primers used in the screening of SNPs by MassArray and PCR direct sequencing. (DOC) [file pone.0021769.s002.doc]

**Table S1.** Details of the primers used in the screening of SNPs by MassArray and PCR direct sequencing.

| SNP | Alleles | Gene | Function | Primers | Extension Primers |
| --- | --- | --- | --- | --- | --- |
| rs12302873* | G/T | PUS7L | intron | TGTGGAGGGGCAGTCATA  ACCAACACCAGCAGTATA | - |
| rs1870765 | A/C | PUS7L | intron | ACGTTGGATGAGTTCATCAGTTCCAGCCAC  ACGTTGGATGGGCAGTGTTTTTTCCTGGTC | CATGGGAATTAAACACAGACCCT |
| rs4251431 | A/C | IRAK-4 | intron | ACGTTGGATGCCCTGGAGATGAAGCATTAG  ACGTTGGATGTGCCTTCCTTCCTCCATTAG | GCATTAGGAAAATTAGAGAAAAAAAA |
| rs4251569 | C/T | IRAK-4 | intron | ACGTTGGATGTCTGGTCACAGTTGGCTTT  ACGTTGGATGGAGGAAAAGATTGTGCAGGG | GCCTGTGCAACCTCAAAGTTCATCTTC |
| rs1461567 | C/T | IRAK-4 | intron | ACGTTGGATGCTAGATAACTAACCCACTCC  ACGTTGGATGAAGGGTAGTTAGGCCATGAG | TAACCCACTCCTGCCATGA |
| rs3794262 | A/T | IRAK-4 | intron | ACGTTGGATGCAGGGTAGAAGTTGAAGATG  ACGTTGGATGGGAAGTTTAGAAGGAAAGAC | CTTACAGCCTAAGCCAGA |
| rs4251481 | A/G | IRAK-4 | intron | ACGTTGGATGCCTGGCCGCAGTCATTATTT  ACGTTGGATGCTCATGAATTCTGGAAGTGG | TGGCCGCAGTCATTATTTTAAGTC |
| rs4251513 | C/G | IRAK-4 | intron | ACGTTGGATGTGCCTATAGGAAGGATCCAGT  ACGTTGGATGGTAACCCTACTGAACACATC | AGATTAAAAGGGAGAGGATA |
| rs4251540 | G/T | IRAK-4 | intron | ACGTTGGATGTGTGTGAACCTCATAGAGT  ACGTTGGATGGGAGCAATACCATATAGCCC | AACCTAGATGATATGACCTAC |
| rs4251559 | A/G | IRAK-4 | UTR-3 | ACGTTGGATGGATACAGTTGGTGGTACAGG  ACGTTGGATGCCTGTTGCCCCTTTCTTTAG | GGTACAGGCAATAAGTAAAACA |
| rs6582484 | C/T | TWF1 | intron | ACGTTGGATGTGGCTTACTAGGAAGTACAG  ACGTTGGATGCTTAGGATCTCCTCTAAGTG | GGGATCATGACAGGTAGT |

*: Genotying by PCR direct sequencing.
