# Supplementary material for: Association Pattern of Interleukin-1 Receptor-Associated Kinase-4 Gene Polymorphisms with Allergic Rhinitis in a Han Chinese Population
Source: PLoS One. 2011 Jun 30;6(6):e21769. doi: 10.1371/journal.pone.0021769 (PMC3128076; doi:10.1371/journal.pone.0021769)
Supplement: Table S2 — SNPs in the IRAK-4 gene selected for genotyping and results of quality testing. (DOCX) [file pone.0021769.s003.docx]

**Table S2**. SNPs in the IRAK-4 gene selected for genotyping and results of basic information

| SNP | HWE *P* | %Geno | MAF |
| --- | --- | --- | --- |
| rs12302873 | 1 | 71.1 | 0.083 |
| rs1870765 | 1 | 100 | 0.02 |
| rs4251431 | 0.1339 | 94.2 | 0.084 |
| rs4251569 | 0.1506 | 100 | 0.155 |
| rs1461567 | 0.2689 | 99 | 0.49 |
| rs3794262 | 0.9322 | 99.6 | 0.143 |
| rs4251481 | 0.6862 | 95.5 | 0.058 |
| rs4251513 | 0.7157 | 99.7 | 0.368 |
| rs4251540 | 0.3653 | 99.4 | 0.084 |
| rs4251559 | 0.0489 | 90.8 | 0.468 |
| rs6582484 | 0.0136 | 96.9 | 0.098 |

HWE: Hardy-Weinberg equilibrium;

%Geno: the nonnull genotype percentage

MAF: minor allele frequency;
