# Supplementary material for: Association Pattern of Interleukin-1 Receptor-Associated Kinase-4 Gene Polymorphisms with Allergic Rhinitis in a Han Chinese Population
Source: PLoS One. 2011 Jun 30;6(6):e21769. doi: 10.1371/journal.pone.0021769 (PMC3128076; doi:10.1371/journal.pone.0021769)
Supplement: Table S3 — MDR analysis summary. (DOCX) [file pone.0021769.s004.docx]

**Table S3**. MDR analysis summary

| No. of loci in model | Model | Testing accuracy | Cross-validation Consistency |
| --- | --- | --- | --- |
| 1 | rs3794262 | 0.5369 | 9/10 |
| 2 | rs4251559, rs3794262 | 0.5559* | 10/10 |
| 3 | rs4251559, rs3794262, rs4251540 | 0.5222 | 4/10 |
| 4 | rs4251559, rs4251569, rs3794262, rs1461567 | 0.4988 | 6/10 |
| 5 | rs4251559, rs4251569, rs3794262, rs4251540, rs1461567 | 0.4919 | 10/10 |

* The overall best MDR model
